# First‐year complications after immediate breast reconstruction with a biological and a synthetic mesh in the same patient: A randomized controlled study

**DOI:** 10.1002/jso.26227

**Published:** 2020-10-13

**Authors:** Emma Hansson, Ann‐Chatrin Edvinsson, Anna Elander, Lars Kölby, Håkan Hallberg

**Affiliations:** ^1^ Department of Plastic Surgery, Institute of Clinical Sciences, The Sahlgrenska Academy Gothenburg University Gothenburg Sweden; ^2^ Department of Plastic and Reconstructive Surgery Sahlgrenska University Hospital Gothenburg Sweden

**Keywords:** acellular dermal matrix, complications, immediate breast reconstruction, synthetic mesh, TIGR, Veritas

## Abstract

**Background:**

Even though meshes and matrices are widely used in breast reconstruction, there is little high‐quality scientific evidence for their risks and benefits. The aim of this study was to compare first‐year surgical complication rates in implant‐based immediate breast reconstruction with a biological mesh with that of a synthetic mesh, in the same patient.

**Methods:**

This study is a clinical, randomized, prospective trial. Patients operated on with bilateral mastectomy and immediate breast reconstruction were randomized to biological mesh on one side and synthetic mesh on the other side.

**Results:**

A total of 48 breasts were randomized. As the synthetically and the biologically reconstructed breasts that were compared belonged to the same woman, systemic factors were exactly the same in the two groups. The most common complication was seroma formation with a frequency of 38% in the biological group and 3.8% in the synthetical group (*p* = .011). A higher frequency of total implant loss could be seen in the biologic mesh group (8.5% vs. 2%), albeit not statistically significant (*p* = .083).

**Conclusions:**

In the same patient, a synthetic mesh seems to yield a lower risk for serious complications, such as implant loss, than a biological mesh.

**Abbreviations**
ASA: American Society of Anaesthesiologist classification
BMI: body mass index
BOADICEA: Breast and Ovarian Analysis of Disease Incidence and Carrier Estimation Algorithm
GA: general anesthetics
LA: local anesthetics
NAC: nipple‐areola complex
NR: not reported
TE: tissue expander

## 1 INTRODUCTION

The usage of acellular dermal matrices was first reported in 2001^1^ and today, a multitude of meshes, both biological and synthetic, have been developed.^2^ Even though widely used,^3^ there is little high‐quality scientific evidence for their risks and benefits.^2^, ^4^

In terms of randomized controlled trials with complications as the primary end‐point (Table 1), there is one study comparing different biological matrices,^5^ two studies comparing biological matrix with traditional muscle cover,^6^, ^7^ and one study comparing biological matrix with synthetic mesh.^8^ The studies suggest that the complication frequencies are higher when biological meshes are used compared with muscle cover.^6^, ^7^ The study comparing biological matrix with synthetic mesh^8^ concluded that there seems to be a higher incidence of implant loss when a biological matrix is used. However, the synthetic mesh used wasnonabsorbable and titanized and therefore constitute an extra permanent foreign material.^8^ The long‐term effects of titanized meshes are unknown. There are no previous randomized controlled studies comparing complication frequencies of a biological mesh with those of an absorbable synthetic mesh.

**Table 1:** Randomized controlled trials on matrices and meshes with complications as primary end‐point

Study	Comparing	No. of breasts	Main results	Implant loss frequency	Frequency of unplanned reoperations for surgical reasons
Biological matrix versus other biological matrix					
Mazari et al^5^	Porcine dermal matrix (Strattice) versus bovine dermal matrix (SurgiMend)	54 versus 43 (67% therapeutic mastectomies)	Bovine dermal matrix seems to have lower frequencies of implant loss, matrix loss, andunplanned re‐operations than porcine dermal matrix. The rates of red breast syndrome and wound infection were higher in the bovine dermal matrix	Porcine dermal matrix: 18.5% Bovine dermal matrix: 7.0%	Porcine dermal matrix: 33.3% Bovine dermal matrix: 7.0%
Biological matrix versus muscle cover					
Dikmans et al^6^ (The BRIOS sudy)	Porcine dermal matrix (Strattice) versus complete muscle/fascial cover	91 versus 92 (64/63% therapeutic mastectomies)	The frequency of complications was markedly higher in the porcine dermal matrix group	Porcine dermal matrix: 11% Muscle cover: 4.3%	Porcine dermal matrix: 32% Muscle cover: 12%
Lohmander et al^7^	Porcine dermal matrix (Strattice) versus complete muscle cover	64 versus 65 (100% therapeutic mastectomies)	There was no difference between the groups as regards the risk for implant loss. There were more wound healing problems requiringreoperation in the porcine dermal matrix group	Porcine dermal matrix: 6% Muscle cover: 6%	Porcine dermal matrix: 17% Muscle cover: 11%
Biological matrix versus synthetic mesh					
Gschwantler‐Kaulich et al^8^	Porcine dermal matrix versus nonresorbable, titanized polypropylene	23 versus 25 (no of patients, no of breasts NR) (60/69.9% therapeutic mastectomies)	The overall complication rates were similar in the two groups. There was a higher incidence of reconstructive failure in the porcine dermal matrix group	Porcine dermal matrix: 30.4% Titanized polypropylene: 7.7%	NR NR

NR, not reported.

The body reacts differently to synthetic and biological meshes. A previous study conducted in our department^9^ demonstrated that there are different histological patterns in early capsules from biological matrices and synthetic meshes. It has been speculated that biological meshes give a better long‐term result and a decreased risk for capsular contracture.^10^ Nonetheless, such theories have not been confirmed in clinical studies, where similar capsular contraction rates have been seen.^11^ In brief, biological and synthetic meshes might cause different tissue reactions but little is known about its significance on complication risks and long‐term results.

We hypothesize that the frequencies of surgical complications are different for absorbable biological and synthetic meshes. The aim of this study was to compare first‐year surgical complication rates in implant‐based immediate breast reconstruction with a biological mesh (Veritas) with that of a synthetic mesh (TIGR Matrix Surgical Mesh), in the same patient.

## 2 PATIENTS AND METHODS

### 2.1 Study design and protocol

This study is a clinical, randomized, prospective trial described in the Gothenburg TIGR/Veritas Study protocol (ClinicalTrials. Gov identifier NCT02985073).

### 2.2 Ethics and informed consent

The Regional Ethical Committee of Gothenburg reviewed and approved the study (189‐16). Procedures followed were in accordance with the Helsinki Declaration of 1964, as revised, and the Good Clinical Practice guidelines. Personal data were treated in accordance with the General Data Protection Regulation. Participants provided written informed consent to participate in the study.

### 2.3 Recruitment of participants

The department is located in one of seven university hospitals in Sweden. All referrals for bilateral immediate breast reconstruction, to our department, were assessed for inclusion. All patients who met the inclusion criteria were asked for participation.

### 2.4 Inclusion and exclusion criteria

Inclusion criteria were 18 years of age or older and indication for bilateral prophylactic mastectomy and immediate breast reconstruction. Exclusion criteria were inability to give informed consent, previous breast surgery, active smoking, and body mass index >30 kg/m^2^. Indication and surgical technique were discussed at a multidisciplinary team conference in all cases.

### 2.5 Sample size

Assuming a target difference in overall complication frequencies between the groups of at least 35%, 32 breasts in each group would be needed to give the study 80% power, for a type I error rate of 5%.

### 2.6 Randomization

The patients were operated with the biological mesh on one side and the synthetic mesh on the other side. During the operation, the patients were randomized, by the sealed envelope method, to which side the biological and the synthetic mesh, respectively, were going to be applied. The design was parallel and the intended allocation ratio in the groups was 1:1. The allocation sequence was concealed and a simple randomization approach was used. The patients were blinded to which mesh they received on which side.

### 2.7 Interventions: Surgical technique and meshes/matrices

The surgical technique has been previously described^12^, ^13^ and was identical in the two breasts, with the exception of the mesh used. In ptotic breasts, a wise pattern skin resection was made, otherwise a submammary incision was performed. All mastectomies were nipple‐sparing. A sub‐pectoral pocket was created and the inferior‐medial and the inferior attachments of the major pectoral muscle were released. The mesh was sutured, with 2‐0 Maxon (Covidien), to the inferior border of the pectoral muscle and to the chest wall corresponding to the inframammary fold and lateral border of the implant pocket; hence a dual‐plane approach was applied. The mastectomies were performed by oncological breast surgeons. All the reconstructions were performed by either HH or EH.

Both meshes used are degradable. Biological Veritas Collagen Matrix (Synovis Surgical Innovations) is a nonfenestrated xenograft made of bovine pericardium. It is composed of non‐cross‐linked propylene oxide‐treated acellular collagen matrix.^14^, ^15^, ^16^ The mesh was not perforated. Human biological meshes are not approved for use in Sweden and were therefore not an option. Synthetic TIGR Matrix Surgical Mesh (Novus Scientific, Uppsala, Sweden) is knitted from two types of fibers; a fast degrading copolymer between glycolide and trimethylene carbonate and a slow‐degrading copolymer between lactic and trimethylene carbonate. The fast degrading part gives extra strength during the healing phase (4 months) and gradually becomes softer and more flexible. The slow‐degrading part is completely resorbed after about three years.^17^ Both meshes have been used in breast reconstruction previously.^12^, ^15^, ^18^, ^19^, ^20^, ^21^, ^22^ An anatomical tissue expander (TE) (CPX, Mentor Worldwide LLC) was used.

During the primary operation two suction drains (Exudrain, Mediplast), French gauge 14, were used for each breast, one subpectoral, and one subcutaneous. The drains were kept in place until the output was less than 30 ml per 24 h for 1 day, but for a maximum of 14 days. Prophylactic perioperative and postoperative antibiotics (cloxacillin or clindamycin, in case of allergy) were given until the drains were removed. During the first two postoperative days a continuous infusion of ropivacaine 2 mg/ml, 4 ml/h, was given in each breast. After drain removal, clinically significant seromas were aspirated superficial to the TE injection port or with the aid of ultrasound. The patients were admitted for 2 days after the stage I operation. The patients wore a compression bra during the first weeks.

The first expander filling was performed after 2–3 weeks in our out‐patient clinic. It was exchanged for a permanent implant (CPG, Mentor Worldwide LLC) about 3 months after the initial operation. Stage II was performed as day‐case surgery.

### 2.8 Clinical data collection

Patient‐related factors, such as age, body mass index, smoking, comorbidities, mastectomy resection weight, and American Society of Anaesthesiology (ASA) grade were recorded. Treatment‐related factors, such as indication for operation, oncologic treatment (neoadjuvant or adjuvant), type of incision, size of TE and implants used, perioperative TE fill, and time between different measures were also recorded.

### 2.9 Outcome: Complications during the first 12 months

All patients were seen 1 and 2 weeks after stage I, during TE fillings, and before the exchange to the permanent implants. They were also seen 1 and 2 weeks and 3 and 12 months after stage II. Adverse events from the time of preparation for anesthesia until 12 months postoperation were registered. Systemic complications that could not be said to be related to one of the breasts were not included in the analysis. The definitions of complications are given in Table 2.

**Table 2:** Definitions of complications

Complication	Definition
Local wound complications	Seroma, red breast syndrome, burn wound in mastectomy flap, delayed wound healing/wound dehiscence, infection, skin necrosis, NAC necrosis
Seroma	A seroma requiring aspiration or leading to that the second stage was brought forward or a significant seroma during the stage II operation (>1 dl)
Red breast syndrome	A noninfectious, self‐limited erythema of the reconstructed breast^28^
Burn wound in mastectomy flap	Burn wounds were defined as clinically visible burn scars at the first follow‐up visit
Infection	Any local surgical site condition requiring treatment with antibiotics. Prophylactic antibiotics given perioperatively or until drains were drawn were not included
Delayed wound healing/wound dehiscence	Any wound healing problems, found on clinical examination, not requiring an intervention in GA or LA. If antibiotics were given the complication was registered as both ‘delayed wound healing’ and ‘infection’. Revision bed‐side were noted separately
Wound revision bed‐side	Partial necrosis of the skin or NAC revised bedside in LA
NAC loss	NAC‐loss that required later NAC reconstruction in LA
Unplanned reoperation	Reoperation in GA due to hematoma, removal of TE/implant, and necrosectomy
Necrosectomy	Necrosis of skin or NAC requiring revision in GA
Hematoma evacuation	Any hematoma requiring surgical exploration
TE loss	A complication that required that the TE was removed. The losses were classified according to the reason for TE loss due to suspect perforation and due to infection
Implant loss	A complication that required that the implant was removed. The implant losses were classified according to reason for implant loss into: due to suspect perforation and due to infection

Abbreviations: GA, general anesthetics; LA, local anesthetics; NAC, nipple‐areola complex; TE, tissue expander.

### 2.10 Statistics

Descriptively, medians and ranges are given, except for daily volume which is given as a mean. Differences between samples from synthetic and biological meshes were analyzed using nonparametric Wilcoxon‐signed rank test for related samples, as the two samples came from the same patient. Analysis was only performed when there was a difference of >2 events between the groups. All tests were two‐tailed and a *p* value of .05 or less was considered statistically significant. All analyses were performed with IBM SPSS Version 25 for Mac (SPSS Inc Chicago).

## 3 RESULTS

Patients were recruited for the study and underwent the stage I operations from 2016 to 2018.

**Figure 1:**
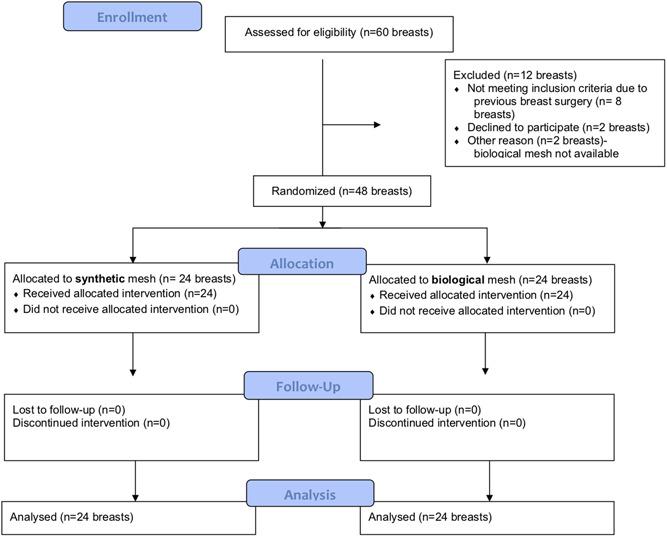
Consort diagram [Color figure can be viewed at wileyonlinelibrary.com]

**Figure 2:**
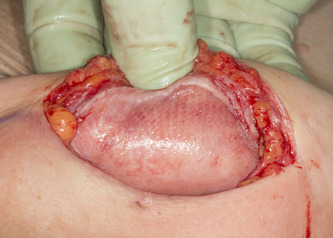
Fully integrated synthetic mesh during the exchange to a permanent implant. Photo: Åsa Bell and Niclas Löfgren, Department of Plastic and Reconstructive Surgery, Sahlgrenska University Hospital [Color figure can be viewed at wileyonlinelibrary.com]

**Figure 3:**
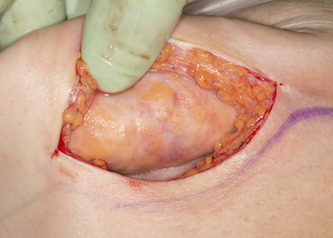
Fully integrated biological mesh during the exchange to a permanent implant. Photo: Åsa Bell and Niclas Löfgren, Department of Plastic and Reconstructive Surgery, Sahlgrenska University Hospital [Color figure can be viewed at wileyonlinelibrary.com]

A total of 48 breasts were randomized, 24 in each group (Figure 1). During the 12‐month visits, it became clear that two meshes yield different esthetic results and therefore an asymmetry. Thus, it became unethical to continue enrolling patients and the recruitment to the study was terminated before 32 breasts had been randomized in each group. No patient was lost to follow‐up and 24 breasts in each group were available for 12 month‐complication analysis (Figure 1). As the synthetically and the biologically reconstructed breasts that were compared belonged to the same woman, systemic factors were exactly the same in the two groups (Table 3). Regarding breast‐related factors (Table 4), there was one known case of invasive tumor in a breast subsequently randomized to a biological mesh, and one case of accidentally found an invasive tumor in breast randomized to synthetic mesh. All other mastectomies were prophylactic. All mastectomies were nipple‐sparing and the majority were performed via an inframammary incision (Table 4). The most common complication was seroma formation with a frequency of 38% in the biological group and 3.8% in the synthetical group (*p* = .011) (Table 5). All synthetic meshes were completely integrated during the exchange to a permanent implant, whereas the biological meshes were poorly integrated in the patients who had seroma (Figures 2, 3). A higher frequency of total implant loss (stage I + II) could be seen in the biologic mesh group (8.5% vs. 2%), albeit not statistically significant (*p* = .083) (Table 5). The two‐implant losses, and re‐operations, related to suspect penetration due to exceptionally thin mastectomy flaps were in the same patient.

**Table 3:** Participants—systemic factors

	Median/number of patients	Range
Age at operation, years	43	25–65
Mutation carriers	19	
Elevated risk but no mutation (according to BOADICEA^a^ calculation)	5	
BMI, kg/m	23.4	19.1–29.6
Active smokers^a^	0	
Previous smokers	4	
ASA 1	21	
ASA 2	3	
Diabetes mellitus	0	
Hypertension	0	
Ischemic heart disease	0	
Obstructive lung disease	1	
Other comorbidities^b^	6	
Neoadjuvant chemotherapy	1	
Adjuvant chemotherapy	0	
Time between stage I and stage II (months)	3.8	(1.4–13.5)
Follow‐up after stage II (months)	16.4	(4.8–40.4)

Abbreviations: ASA, American Society of Anaesthesiologists classification; BMI, body mass index; BOADICEA, Breast and Ovarian Analysis of Disease Incidence and Carrier Estimation Algorithm.^29^

a The patients were required to stop smoking 6 weeks before the operation.

b Reflux, inflammatory bowel disease, depression, arthrosis, arrythmia, liver fibrosis, irritable bowel disease.

**Table 4:** Participants—breast‐related factors

Breast‐related factors	Biological (median (range) or number of patients)	Synthetic (median (range) or number of patients)
Resection weight, g	267.5 (68–616)	267.5 (77–597)
Risk reduction	23	23
Breast cancer	1	1^a^
Nipple‐sparing mastectomy	24	24
Inframammary incision	20	20
Wise pattern incision	4	4
Axillary surgery	1^b^	1^a^
TE size, ml^c^	350 (250–450)	350 (250–450)
TE fill during operation, ml	100 (0–250)	100 (0–250)
Fill ratio, %	30 (0–72)	30 (0–72)
Size permanent implanted, cm^3^	350 (100–440)	350 (100–440)
Pre/postoperative radiation	0	0

Abbreviations: Op, operation; TE, tissue expander.

a A small invasive breast cancer was found in the resection. Axillary sampling was performed in a separate operation.

b Sentinel node biopsy.

c 46 breasts—24 in each group as one patient received permanent implants during the first operation

d 48 breasts. Hence the patient who received permanent implants during the first operation has been included here. Three patients (six breasts) received round implants according to their own wish (Siltex Mentor Worldwide LLC).

**Table 5:** Surgical site complications in the two study groups

	Biological	Synthetic	*p* value
Stage I	*n* = 24	*n* = 24	
Local wound complications			
Seroma	9 (38%)	2 (8.3%)	.011
Red brest syndrom	0	0	
Burn wound in mastectomy flap 0		2 (8.3%)	
Delayed wound healing/wound dehiscence (not requiring bed‐side revision)	2 (8.3%)	1 (4.2%)	
Infection	3 (12.5%)	0	.083
Skin necrosis	0		
NAC necrosis	0	0	
Unplanned re‐operations for surgical reasons			
Hematoma evacuation	0	1 (4.2%)	
Wound revision in LA	1^c^ (4.2%)	0	
Wound revision in GA	1^c^ (4.2		
Expander loss due to suspect perforation	1^a^ (4.2%)	1^a^ (4.2%)	
Expander loss due to infection	2 (8.3%)	0	
Stage II	*n* = 23^b^	*n* = 23^b^	
Local wound complications			
Seroma	0	0	
Red brest syndrom	0	0	
Delayed wound healing/wound dehiscence	0	0	
Infection	1 (4.3%)	0	
Skin/NAC necrosis	0	0	
Unplanned re‐operations			
Hematoma evacuation	0	0	
Necrosectomy	0	0	
Implant loss due to suspect perforation 0		0	
Implant loss due to infection	1 (4.3%)	0	
Stage I + II	*n* = 47^b^	*n* = 47^b^	
Unplanned re‐operations for surgical reasons	*4 (8.5%)*	*2 (4.3%)*	
TE/implant loss	4 (8.5%)	1 (2%)	.083
Hematoma evacuation	0	1 (2%)	

a Implant was lost on both sides in the same patient.

b One patient received permanent implants during the first operation.

c The same breast was revised once in LA and once in GA.

## 4 DISCUSSION

The present study investigates differences between a biological and a synthetic mesh regarding complication frequencies during the first 12 months after surgery. It is the first study to randomize between the two breasts in the same individual. The findings indicate that there is a higher risk for complications, particularly seroma and implant loss, when a biological mesh is used.

Risk factors for complications in immediate breast reconstruction include aspects related to the patient, to the surgical procedure, and to the oncological treatment.^23^ A major strength of the present study is that the biological and synthetic mesh was used in the same patient. That is, patient‐related factors, such as comorbidity, previous smoking, body mass index, breast size, and age,^23^, ^24^, ^25^ were exactly the same for both materials. Similarly, surgical factors, such as the surgeon′s experience,^25^, ^26^ TE/implant type and size, number of stages,^27^ and type of incision used^28^ were identical in the two groups as the procedures were performed by the same surgeons and the techniques were the same in both breasts. In addition, the two reconstructive surgeons who performed the reconstructions have comprehensive experience with breast reconstruction using meshes,^12^, ^13^ so there should not have been a learning curve affecting the results. Only two patients received systemic oncological treatment and it was of course the same for both breasts. None of the patients received or had received radiotherapy. In conclusion, conditions affecting complication risks were identical in the two groups, which is a unique strength of the present study.

Previous randomized controlled studies have indicated that the risk for implant loss is higher when a biological mesh is used, compared with traditional muscle cover^6^ and compared with titanized nonabsorbable synthetic mesh^8^ (Table 1). Our results are in accordance with these findings and indicate that the frequency of implant loss is higher in the biological mesh group (Table 5). If we look at the implant losses caused by an infection in the present study, three breasts (3/24, 12.5%) were affected in the biological mesh group and none in the synthetic mesh group. It has been hypothesized that the higher infection rates seen in biological meshes are secondary to seroma and that a higher postoperative seroma incidence is directly associated with the biological meshes.^29^, ^30^, ^31^ Indeed, there was more seroma formation in the biological mesh group (Table 4). Given our results, it could also be hypothesized that the susceptibility to implant loss somewhat remains after stage II, as one implant was lost after stage II (Table 5). In summary, the results of the present study support that there is a higher incidence of seroma, secondary infection, and implant loss when biological meshes are used.

Although this a randomized controlled trial with identical background factors in the two groups, it has a few methodological weaknesses. Firstly, it was terminated as it became clear that the two meshes yield different esthetic results and therefore an asymmetry. The esthetic results, and benefits and drawbacks with the two meshes in that respect, will be analyzed in a separate study. The plastic surgeons involved judged that it would be unethical to continue enrolling patients, knowing that a large proportion of them might need esthetic corrections in the long run, and we therefore chose to possibly “sacrifice” statistically significant results regarding complication frequencies. Nonetheless, clear clinical differences could be seen between the groups (Table 5), albeit not statistically significant. Secondly, some of the complications are still rare, although more common in the biological group. The sample size was calculated based on total complication frequencies and much larger samples would probably be required to establish differences in individual complications between groups, with high certainty. Thirdly, the study compares a single biological mesh with a single synthetical mesh. Hence, the results might be biased by individual qualities of the meshes that are unrelated to whether they are biological or synthetical. Even so, as regards first‐year complication frequencies, the safety of the meshes used in the present study has been demonstrated previously.^12^, ^18^ Therefore, there is no suspicion that any of the meshes used are of lower quality than other meshes currently on the market.

In conclusion, in the same patient, a synthetic mesh seems to yield a lower risk for serious complications, such as implant loss, than a biological mesh. Although very important, other factors such as costs and long‐term esthetic and functional results, also need to be considered when the type of mesh is chosen. A longer follow‐up of the patients will be performed in the future to address those factors.

## CONFLICT OF INTERESTS

The authors declare that there are no conflict of interests.

## SYNOPSIS

This study is a clinical, randomized, prospective trial where patients operated on with bilateral mastectomy and immediate breast reconstruction were randomized to biological mesh on one side and synthetic mesh on the other side. In the same patient, a synthetic mesh seems to yield a lower risk for serious complications, such as implant loss, than a biological mesh.
